# Anisotropy and Mechanical Properties of Nanoclay Filled, Medium-Density Rigid Polyurethane Foams Produced in a Sealed Mold, from Renewable Resources

**DOI:** 10.3390/polym15112582

**Published:** 2023-06-05

**Authors:** Ilze Beverte, Ugis Cabulis, Janis Andersons, Mikelis Kirpluks, Vilis Skruls, Peteris Cabulis

**Affiliations:** 1 Institute for Mechanics of Materials, University of Latvia, 3 Jelgavas St., LV-1004 Riga, Latvia; ilze.beverte@lu.lv (I.B.); janis.andersons@pmi.lv (J.A.); skruls@pmi.lv (V.S.); 2 Latvian State Institute of Wood Chemistry, 27 Dzerbenes St., LV-1006 Riga, Latvia; mikelis.kirpluks@kki.lv (M.K.); peteris@ritols.lv (P.C.)

**Keywords:** rigid polyurethane foams, medium density, nanoclay, sealed mold, mechanical properties, monotropy, Poisson’s ratios

## Abstract

Medium-density rigid polyurethane (PU) foams are often produced in sealed molds; therefore, the processes inside the mold and structure of the produced foam blocks need to be understood. The structural and mechanical anisotropy is shown to be the third variable along with (1) concentration of the nanoclay filler and (2) density, to determine the mechanical properties of the filled PU foam composites produced in a sealed mold. The varying anisotropy of the specimens hinders the accurate evaluation of the filling effect. The methodology for the estimation of the anisotropy characteristics of specimens from different locations within the nanoclay filled PU foam blocks is elaborated. A criterion, based on analysis of Poisson’s ratios, is formulated for the selection of specimens with similar anisotropy characteristics. The shear and bulk moduli are estimated theoretically, dependent on the filler’s concentration, using the experimentally determined constants.

## 1. Introduction

Rigid polyurethane (PU) foams of medium apparent density ~200–250 kg/m^3^ are widely applied as a structural material in various engineering solutions, especially in the automotive industry for test milling, design studies and modelling; as substructures for model pastes; making of simple negative molds and laminating molds; for impact absorption, etc. [1,2,3,4]; as encapsulants for electronic components to mitigate harsh thermal and mechanical environments and to provide electrical isolation [5,6], etc.

In PU foam production, one sustainable solution is the use of recycled materials instead of petrochemical raw materials. The poly(ethylene terephthalate) (PET) manufacturing by-product provides an appropriate resource for aromatic polyester polyols. PU foams obtained from these polyols have better mechanical and thermal properties because of the introduction of the aromatic structure into the PU polymer matrix [7,8,9,10].

Nanoclay, such as montmorillonite (MMT), has become a popular nanofiller in many polymeric systems as it imparts characteristics such as light weight, improved thermal stability, flame retardancy, and high compressive strength [9,11,12,13,14]. However, the hydrophilic nature of MMT causes a weak interfacial adhesion with the polymer matrix which is hydrophobic [14]. Modification of the MMT is needed in order to enhance the compatibility and dispersibility of the MMT in the polymer matrix, thus improving the load transfer efficiency of the system [14,15]. The interface between the filler and the polymer matrix in the nanocomposites constitutes a larger area than in ordinary composites based on micrometer-sized fillers; therefore, the interface impacts the properties of the nanocomposites to a higher degree [12,15].

Filling PU foams with nanoclays can provide valuable improvements and modifications in the properties [9,11,12,13,14,15,16,17,18,19]. Well-dispersed nanoparticles act as nucleation sites, thus facilitating bubble formation and leading to a reduction in the cell size of foams [11,14]. The exfoliated clay nanoplatelets in the cell walls reduce gas diffusivity (the barrier effect) and enhance the mechanical properties of the foams [13,14,16]. At the same time, at higher clay loading, an excess of nanoclay content may result in the agglomeration or clustering of the clay [14]. The efficiency of mechanical reinforcement is much determined by the degree of dispersion, intercalation, and/or exfoliation of the filler [14,15]. Depending on the chemical structure of polyurethane, as much as a 650% increase in reduced compressive strength was observed in a PU nanocomposite foam with a relatively low cross-linking density and urethane content but the opposite effect was observed in PU nanocomposite foams with a highly cross-linked structure and high urethane content [11].

The improvements provided by the nanofiller reinforcing agents in free-rise PU foams are often accompanied by changes in the PU foams’ density, where density monotonically decreases as the clay content increases due to the additional blowing by the bound water [17,18,20]. The density of specimens may also differ for foams produced in sealed molds [9]. However, the efficiency of nanofillers can only be judged once the density differences are accounted for. One solution may be to normalize the mechanical property by the foams’ density, e.g., by comparing the specific strength or specific modulus [11].

The mechanical properties of polymeric foams are plotted against the foams’ density, then normalization to a common density is performed [1,5,9,20,21]. The structural anisotropy of low- and medium-density PU foams is determined by the different spatial orientations of the structural elements (polymeric struts, walls, gaseous cells) relative to the axes of the external coordinate system. When neat (unfilled) and nanoclay filled PU foams are produced in an open mold of equal transversal dimensions, the anisotropy mode of the foams is monotropy. The degree of monotropy of filled PU foams differs from that of neat foams, since the foams rise to different heights [1,22,23]. As a result, the mechanical properties of the neat and filled PU foams differ not only due to the filling, but also because of different monotropy degrees. An accurate evaluation of the impact of filling on the mechanical properties is hindered by differing structural anisotropy; therefore, open molds are not suited for such studies. 

With the proper choice of technological parameters, foaming in a sealed mold permits the production of nearly isotropic PU foams, thus reducing the influence of the anisotropy variations. In [5], a mixed liquid composition of PU foams was poured into cylindrical molds at room temperature to produce PU foams of density 100–400 kg/m^3^. The molds were then closed and the foam was allowed to expand to fill the closed molds at a packed density of approximately 1.75 times the expected free-rise density. In [6], the molds used for foaming PU foams CRETE and epoxy foams consisted of two steel plates, perforated with small holes to allow gas and foam escape, with steel cylinders of various heights and diameters between the plates. In [12], the liquid reacting mixture of PU foams, with an expected density of 240 kg/m^3^, was poured into a preheated aluminum mold, made as two aluminum plates on both sides of an aluminum frame screwed together with four steel rails and bolts. Closed plastic containers were used for the foaming [11]. In [9,10], the reacting mixture of NEOpolyol-380 PU foams was poured into a stainless steel mold and the mold was sealed.

The main aim of this study was to increase the estimation accuracy of the effect of filling rigid PU foams with nanoclay on the foams’ mechanical properties, when the foams are produced in a sealed mold. The methodology for the selection of nanoclay filled PU foam specimens of similar anisotropy characteristics was elaborated. The adjustment caused by the selection of specimens on the increase/decrease in the mechanical properties of the filled PU foams was evaluated. It was concluded that the selection of specimens of similar anisotropy characteristics reduced the influence of anisotropy variations, especially for the reference material—the neat PU foams—and thus, the accuracy of estimation of the filling effect on the mechanical properties was increased.

## 2. Materials and Methods

### 2.1. Raw Materials

The aromatic polyester polyol (APP) NEOpolyol-380 used in the given investigation was produced by Neo Group, Rimkai, Lithuania. The company produces mainly PET granules and PET bottles. The by-product of those commodities comprises PET dust and other industrial waste, which is directly transferred into a glycolysis reactor where it is converted into APP. A higher functional polyether polyol based on sorbitol Lupranol 3422 (contains only secondary hydroxyl groups, OH value 490 mg KOH/g) from BASF, Ludwigshafen, Germany, was added to increase the cross-linkage density of the polymer matrix. An additive surfactant NIAX Silicone L6915 was used to obtain closed-cell PU foams. The reactive delayed action time amine-based catalyst NP-10, available from Momentive Performance Materials Inc., Niskayuna, NY, USA, was used. Tris(chloropropyl)phosphate (TCPP) from LANXESS Deutschland GmbH, El Dorado, AR, USA, was used as a flame retardant. Water acts as a chemical blowing agent and releases carbon dioxide CO_2_ in reaction with isocyanate. Polymeric diphenylmethane diisocyanate—IsoPMDI 92140 (pMDI) from BASF, Ludwigshafen, Germany, —was used as an isocyanate component (NCO = 31.5 wt.%).

Industrially produced MMT nanoparticles Cloisite-30B (Manufacturer BYK Additives, Louisville, KY, USA; year 2010 production) were used as the filler. Cloisite-30B is organically modified with organic modifier methyl, tallow, bis-2-hydroxyethyl, quaternary ammonium; specific gravity 1980 kg/m^3^. The size distribution of clay agglomerates prior to dispersion was ~50% ≤ 6 μm (6000 nm) and ~90% ≤ 13 μm (13,000 nm), the X-ray diffraction d-spacing (001) 18.5 Å.

### 2.2. Production of NEOpolyol-380 PU Foams in a Sealed Mold

**Figure 1:**
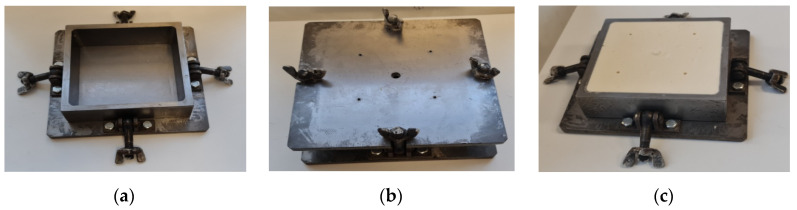
The mold (**a**) open, (**b**) with a sealed lid, and (**c**) with a PU foam block inside.

Rigid polyurethane (PU) foams blocks shaped as a truncated pyramid, top 15 × 15 cm, bottom 14 × 14 cm, height 5 cm and inner volume 0.00105 m^3^, were produced in a sealed steel mold, Figure 1. The polyol component was made by weighing the components (he recycled polyol NEOpolyol-380, cross-linking agent Lupranol 3422, flame retardant, blowing agent, catalyst and surfactant) and stirring them for 1 min with a mechanical stirrer at 2000 rpm.

The pMDI and polyol components were weighed and mixed with a mechanical stirrer at 2000 rpm for 15 s. The mold was preheated to 50 °C and an appropriate amount of the reacting mixture was poured into it; then, the mold was sealed. The mass of the reacting mixture was calculated in order to obtain PU foams with an approximate apparent overall density of 250 kg/m^3^.

The mixed-in air and gases of the chemical reactions escaped from the mold through 4 peripheral and 1 central gas-release holes (PGRH-s and CGRH). The CGRH was closed after all air/gases had escaped and the foaming mixture started to come out. The PGRH-s were left open and the foams sealed them by coming out and hardening. Then the PU foams were cured at 50 °C for 2 h. The mold was cooled to room temperature; PU foam block was removed and conditioned for at least 24 h.

In a sealed mold, where overpressure occurs, the PU foams’ gaseous cells are more spherical than ellipsoidal, so that an isotropic structure is obtained. In a study on sandwich panel manufacturing, a sealed mold and overpressure was used to obtain an isotropic PU foam core [24]. The closeness of the PU foams’ structure to isotropic one depends on how high the overpressure is. In sandwich panels manufactured at a lower overpressure, an anisotropic structure can be observed [25].

The PU foams’ formulation is given in Table 1.

The formulation comprised ~15% of recycled materials. The recycled APP NEOpolyol-380 can be considered a sustainable raw material, because it is produced from industrial PET waste. Nanoclay Cloisite-30B was added as a filler in concentrations η = 0.00, 0.25, 0.50, 1.00, 2.00, 3.00 and 5.00% of the mass of the filled reacting mixture by mixing the filler into the NEOpolyol-380 with a high shear mixer Silverson, East Longmeadow, MA, USA, for 20 min at 8000 rpm.

The mass of the liquid reacting mixture, poured into the mold, was calculated so as to make PU foam blocks of apparent overall density ≈250 kg/m^3^ (skins included; ISO 845:2006). The technological target was to keep the mass of the filled reacting mixture constant for all 7 concentrations: m_0_ = 250 g = const. Foaming in a sealed mold, with counter-pressure, is supposed to provide PU foams of nearly isotropic structure.

The content of closed cells in neat PU foams was measured (ISO 4590:2016). Density of neat, free-rise PU foams of the given formulation, see Table 1, was determined, producing foams in an open mold of the same transversal dimensions as those of the sealable mold. The value of overpressure in the sealed mold was calculated as p_ov_ = *ρ*_sm_/*ρ*_0_, where *ρ*_sm_ is the apparent overall density of a block produced in a sealed mold and *ρ*_0_ is the same for a block produced in an open mold.

### 2.3. XRD Analysis

When nanoclay is filled into a monolithic polymer or plastic foam, the mechanical properties of the composite are determined by the degree of dispersion, intercalation, and/or exfoliation of the filler. Intercalation and exfoliation of Cloisite-30B monolayers were evaluated via the basal spacing by X-ray diffraction (XRD), at a 5 wt.% concentration of nanoclay (from the mass of NEOpolyol-380) in “Cloisite-30B-NEOpolyol-380” dispersions. The dispersions were made by (a) high shear mixing with a mixer Silverson, East Longmeadow, MA, USA, the effective mixing time 20 min at 8000 rpm and (b) sonication, the effective sonication time 20 min. An Ultrasonic Cell Crusher, MRC, Holon, Israel, was applied over short periods, frequency 20–25 Hz, 5 s of active period and 5 s of passive period to reduce heating of the mixture. Temperature limit was set to 40 °C and was controlled by a water bath. At both dispersion modes, milky, homogenous, and stable dispersions were produced.

The “Bruker D8 Discover” diffractometer, Bruker AXS GmbH Karlsruhe, Germany, was applied to obtain XRD patterns of the “Cloisite-30B-NEOpolyol-380” dispersions. The diffractometer comprised a “LynxEye” detector, operated in 0D-mode and a copper radiation source (CuKα), operated at a wavelength λ = 0.15418 nm. The tube voltage of the diffractometer was set to 40 kV and the current to 40 mA. The width of the divergence slit was 0.2 mm and that of the anti-scattering slit 8.0 mm. An appropriate amount of dispersion was spilled into holders, which were rotated during measurement. Registration of the XRD patterns was made at a speed of scan 10 s/0.01° from 1.5 to 7 degrees (in the 2θ scale).

### 2.4. NEOpolyol-380 PU Foams’ Blocks and Specimens

**Figure 2:**
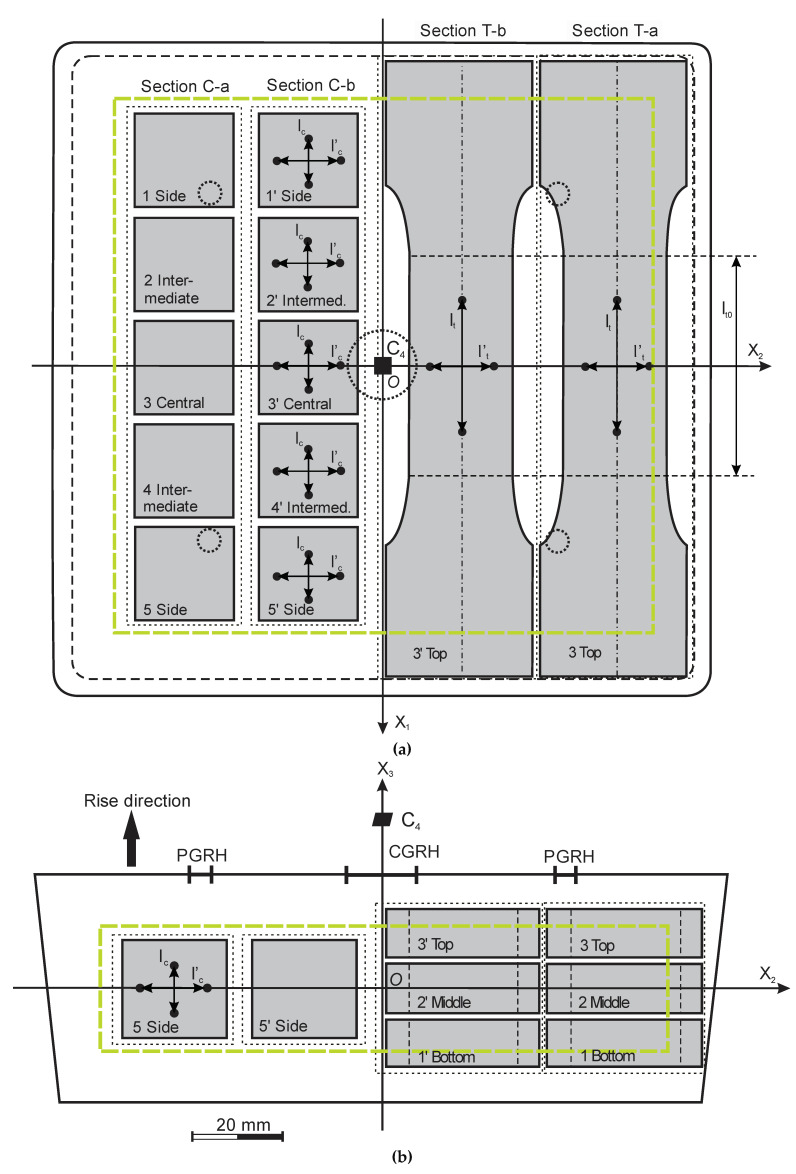
A PU foam block: (**a**) top view and (**b**) side view; the green rectangles enclose a zone of highly uniform density; C-a and C-b are sections of compression specimens, T-a and T-b are sections of tension specimens. X_1_OX_2_X_3_—a coordinate system, associated with the block (CGRH—the central gas-release hole and PGRH-s—the peripheral gas-release holes).

The mass of the 7 NEOpolyol-380 PU foam blocks was determined. Each block was divided into four sections: C-a and C-b for compression specimens; T-a and T-b for tension specimens, see Figure 2, where the foams’ rise direction was parallel to the axis OX_3_ and X_1_OX_2_ was the plane of isotropy. Molding skins, thickness ~8 … 10 mm, were cut off prior to making the specimens.

Five compression specimens shaped as cubes of dimensions 22 × 22 × 22 mm were made from each section C-a and C-b and their apparent core density (ISO 845:2006, further density) was determined. Three parallelepipeds, size ≈ 10 × 33 × 140 mm, precursors of tension specimens, were made from each section T-a and T-b and their density was determined. A dumbbell shaped tension specimen with a straight work zone of dimensions l_t0_ ≈ 10 × 24 × 50 mm was made from each precursor. Prior to the test, the work zone was marked on the tension specimens and its dimensions were measured. After testing, the work zone elements were cut from the ruptured specimen, weighed and initial density in the work zone was calculated.

The compression specimens were made as cubes in order to measure the lateral and transversal displacements simultaneously, on one and the same side of a specimen. The values of the displacements allowed us to calculate the Young’s moduli and Poisson’s ratios in compression (1) parallel and (2) perpendicular to the foams’ rise direction. The size of specimens was chosen so as to have them located in the zone of uniform density of the PU foam blocks, because the compressive response of PU foams is more sensitive to nonhomogeneous density than the tensile response. The tension specimens were made with their longitudinal axis parallel to the axis OX_1_, Figure 2; thus, the elasticity modulus E_1_, Poisson’s ratio ν_12_ and strength σ_11_ were determined both in compression and tension and a comparison could be made.

### 2.5. Density

#### 2.5.1. Gradient of Density

To estimate gradient of density *ρ*’ = δ*ρ*/δx_2_ along axis OX_2_ in the sections C-a and C-b, their density was determined prior to making compression specimens. The relative difference in densities was calculated as R = Δ*ρ*/*ρ*_C-a_ = (*ρ*_C-a_ − *ρ*_C-b_)/*ρ*_C-a_, where *ρ*_C-a_ and *ρ*_C-b_ are density of the sections. Additional estimation of NEOpolyol-380 foams’ structure at the sides of the blocks was made with a light microscope Diamond MCXMP500, MICROS Produktions-& Handels GmbH, Sankt Veit an der Glan, Austria, on thin layers of foams, in transmitted lighting, magnification 10×.

#### 2.5.2. Density of Compression and Tension Specimens

The locations of the compression specimens in the NEOpolyol-380 PU foams’ blocks were denoted as “Side” (1, 1′ and 5, 5′), “Intermediate” (2, 2′ and 4, 4′) and “Central” (3, 3′), Figure 2. The average density, standard deviation and coefficient of variations were estimated for specimens from the sections C-a and C-b. The densities of specimens from similar locations 1, 1′; 2, 2′; 3, 3′; 4, 4′ and 5, 5′ in the sections C-a and C-b were compared to estimate the density distribution along the axis OX_2_. Density distribution along axis OX_1_ was estimated, comparing densities of specimens 1 … 5 and 1′ … 5′.

The locations of the tension specimens in the blocks were denoted as “Bottom” (1, 1′), “Middle” (2, 2′) and “Top” (3, 3′), Figure 2. The average density, standard deviation and coefficient of variations were estimated for (1) the precursors and (2) the straight part of the tension specimens. Densities of (1) the precursors and (2) the straight part of the tension specimens from similar locations 1, 1′; 2, 2′ and 3, 3′ in the sections T-a and T-b were compared to estimate the density distribution along the axis OX_2_. Density distribution along axis OX_3_ was estimated, comparing densities of specimens 1–3 and 1′–3′. Density distribution along axis OX_1_ was estimated, comparing densities of the precursors and of the straight part.

Densities of compression specimens from locations 3 and 3′ were compared with the densities of tension specimens from locations 2 and 2′, located side by side in a block, symmetrically to the plane X_1_OX_3_, at a similar coordinate OX_3_ (Figure 2).

### 2.6. Mechanical Testing

The mechanical response of NEOpolyol-380 PU foams was tested in compression and tension according to the main principles of ISO 844:2021 and ISO 1926:2009. Stress–strain curves were registered on a testing machine Z-100 TEW, Zwick GmbH & Co. KG, Ulm, Germany, with a video extensometer videoXtens 2-120 HP and a camera uEye 01-3483CP-M-GL. The five compression specimens of the section C-a were loaded parallel to the rise direction OX_3_ and those of the section C-b, perpendicular to the RD (parallel to axis OX_1_), Figure 2. The 3 + 3 = 6 tension specimens of both sections T-a and T-b were loaded perpendicular to the RD (parallel to axis OX_1_).

Displacement was measured parallel to the loading direction: (a) in compression on a base l_c_ = 10 mm and (b) in tension on a base l_t_ = 30 mm, as well as in the transversal direction on bases l_c_’ = l_t_’ = 15 mm in compression and in tension, Figure 2. The crosshead speed was 10%/min (2.2 mm/min in compression and 5 mm/min in tension); ambient temperature ≈ 23 °C. Because of the limited height of the mold, the compressive specimens had similar dimensions in the transversal and lateral directions and a homogeneous compressive stress field [1] could not be ensured in the measurement zone. Therefore, the calculated compression modulus E_1_ (E_3_) was considered as stiffness of the specimen in compression parallel to axis OX_1_ (OX_3_).

Moduli E_1_, E_3_, Poisson’s ratios ν_12_ = −Δε_22_/Δε_11_, ν_32_ = −Δε_22_/Δε_33_ in compression and modulus E_1_ and Poisson’s ratio ν_12 =_ −Δε_22_/Δε_11_ in tension were determined from the initial linear region of the stress–strain curves. Compressive stress at 10% strain σ_11(10%)_ and σ_33(10%)_ was determined as compressive force at 10% strain, divided by the initial cross section of the specimen; ISO 844:2021(E). In tension, strength σ_11max_ and elongation at break ε_11max_ was determined from the stress–strain curves at the maximum force. The remaining strain of compression specimens was measured ~1.5 years after testing.

### 2.7. Poisson’s Ratios and Density

Poisson’s ratios of cellular plastics depend on structural anisotropy and do not depend directly on density [4,22,23]. To examine it closely, light-weight, highly homogeneous, monotropic, neat PU foams of a standard petrochemical formulation, produced in a free-rise, of different densities <90 kg/m^3^, were ordered from an industrial-scale production enterprise.

Five blocks of core foams, size 50 × 50 × 20 cm, were supplied. The foams were tested in compression and tension according to the main principles of ISO 844:2021 and ISO 1926:2009. To ensure a homogeneous stress field in the measurement zone, the compression specimens were made as rectangular prisms of dimensions 100 × 50 × 50 mm, strain rate 10%/min. The tension specimens were made dumbbell shaped, dimensions of the straight part 20 × 25 × 55 mm, strain rate 10%/min. Displacement was measured parallel to the loading direction, base 30 mm, both in compression and tension as well as in the transversal direction, bases 50 mm in compression and bases 25 mm and 20 mm in tension. Electromechanical testing machine Z-100 TEW, Zwick GmbH & Co. KG, Ulm, Germany, was used for mechanical testing. Four specimens were tested for each data point; the ambient temperature was T = +23 °C. Density of specimens was determined prior to testing.

Moduli E_1_ and E_3_, Poisson’s ratios ν_12_, ν_13_, ν_31_ and ν_32_ were determined from the initial, linear region of the stress–strain curves, which were registered for loading in compression parallel and perpendicular to the foams’ rise direction and in tension parallel to the rise direction. Strength σ_11max_, σ_33max_ and the corresponding strain ε_11max_, ε_33max_ in compression as well as strength σ_33max_ and elongation at break ε_33max_ in tension were determined from the stress–strain curves.

## 3. Theoretical Section

### 3.1. The Criterion of Similar Anisotropy Characteristics

The structure and elastic properties of PU foams often exhibit anisotropy of a certain mode as orthotropy or monotropy [1,2,4,22,23]. Foams produced in an open mold of equal transversal dimensions are monotropic [26,27,28,29]:

E_3_ ≥ E_1_ = E_2_; ν_32_ = ν_31_ ≥ ν_12_; σ_33max_ ≥ σ_11max_ = σ_22max_. (1)

The degree of monotropy, DM, can be characterized by the ratio of (1) moduli E_3_/E_1_ or E_3_/E_2_; (2) Poisson’s ratios ν_31_/ν_13_ or ν_32_/ν_23_; (3), average projections of cells’ diameters d_3_/d_1_ or d_3_/d_2_; (4) the average projections of polymeric struts’ length l_3_/l_1_ or l_3_/l_2_, etc. [1,23,30]. The bigger the height to width ratio of the open mold, the higher the degree of monotropy in the produced foams. Upon reaching the lid, the liquid reacting mixture foaming proceeds similar to a free-rise and the foams are expected to be monotropic/isotropic in a mold with a square cross-section.

The properties of the NEOpolyol-380 PU foams in a block are determined by (1) symmetry of the mold in the horizontal plane X_1_OX_2_, (2) the limiting dimensions of the lab-scale mold, especially height, (3) cooling at the sides, top and bottom of the mold due to non-adiabatic processes, (4) the different rise speeds of the foams in the centre and at the sides of the mold, etc. The specimens occupy different locations in the block relative to the factors mentioned that influence their anisotropy characteristics. Thus, anisotropy is the third variable along with (1) the concentration of the filler and (2) density, determining the mechanical properties of PU foams. Simultaneous variation of several of the foams’ characteristics does not permit correct estimation of the dependence of the mechanical properties on the concentration of filler.

It was proposed to reduce the influence of anisotropy variations by selecting specimens with similar characteristics of anisotropy. Since Poisson’s ratios of cellular plastics depend on structural anisotropy and do not depend directly on density [4,22,23], a criterion, based on the analysis of the Poisson’s ratios, was formulated for the selection of specimens of similar anisotropy characteristics: mode and degree. For NEOpolyol-380 PU foams, produced in a mold of equal transversal dimensions (Figure 1 and Figure 2), the anisotropy mode is monotropy/isotropy and the degree of monotropy DM is estimated by the ratios of Poisson’s ratios:

DM = ν_31_/ν_13_ or DM = ν_32_/ν_23_. (2)

The values of Poisson’s ratios of NEOpolyol-380 PU foam specimens had to be analyzed to select specimens with similar anisotropy characteristics. Three experimental data sets of Poisson’s ratios were analyzed: (1) ν_12_ in compression (N_1_ = 7 blocks × 5 specimens = 35 specimens), (2) ν_32_ in compression (N_2_ = 7 × 5 = 35 specimens) and (3) ν_12_ in tension (N_3_ = 7 × 3 + 7 × 3 = 42 specimens) and average values were calculated:

(3) $$\nu {12_{\mathrm{av}}}^{C}=1/N_{1}\;\sum_{n=1}^{N1}\nu_{12n}^{C},\;\nu {32_{\mathrm{av}}}^{C}=1/N_{2}\;\sum_{n=1}^{N2}\nu_{32n}^{C}\;\mathrm{and}\;\nu {12_{\mathrm{av}}}^{T}=1/N_{3}\;\sum_{n=1}^{N3}\nu_{12}^{T}$$

For each value of ν_12n_^C^, ν_32n_^C^ and ν_12n_^T^, the relative difference from the corresponding average was calculated:

R1_n_ = (ν_12av_^C^ − ν_12n_^C^)/ν_12av_^C^, n = 1, 2, …, N_1_; R2_n_ = (ν_32av_^C^ − ν_32n_^C^)/ν_32av_^C^, n = 1, 2, …, N_2_ and

R3_n_ = (ν_12av_^T^ − ν_12_^T^)/ν_12av_^T^, n = 1, 2, …, N_3_. (4)

A criterion of similar anisotropy characteristics was formulated: the absolute value of the relative difference between the Poisson’s ratio of a specimen and the average value of the corresponding experimental data set must not exceed a predefined limit:

∣R1_n_∣ ≤ δ_1_; n = 1, 2, …, N1, ∣R2_n_∣ ≤ δ_2_; n = 1, 2, …, N2 and ∣R3_n_∣ ≤ δ_3_; n = 1, 2, …, N3, where δ_1_, δ_2_, δ_3_-parameters (5)

The values of the parameters were set to δ_1_ = δ_2_ = δ_3_ = 0.1 (10%). To test the selected data, the average values and relative differences were calculated for the selected data sets and their elements were checked again for fulfilling the criterion, now with the recalculated average values and relative differences. The experimental data of the specimens, whose Poisson’s ratios fulfil the criterion, were selected for further processing (normalizing, averaging, etc.); the others were excluded. If such data was detected, which do not fulfill the criterion, the selection procedure.

### 3.2. Normalizing of the Mechanical Properties to a Common Density

For each concentration of filler, there were three groups of NEOpolyol-380 PU foam specimens: (1) five specimens for compression parallel to axis OX_1_, (2) five specimens for compression parallel to OX_3_ and (3) six specimens for tension parallel to OX_1_. When, in each group, the mechanical properties and density were averaged over the selected specimens, different average densities were acquired, which made comparison of the mechanical properties inaccurate. Therefore the mechanical properties had to be recalculated to a certain common density (normalized).

An empirical relationship exists between a modulus (or strength) and the density of rigid, isotropic, neat PU foams [1,4].

E = A*ρ*^b^, where 1.0 ≤ b ≤ 2.0. (6)

The parameters A and b depend on several factors: (1) the formulation of the polyurethane matrix, (2) the type and concentration of filler, (3) the foaming mode: free-rising (anisotropic foams) or in a sealed mold (isotropic foams), (4) the loading mode: compression or tension, since PU foams often exhibit different mechanical properties in compression and in tension, even in the elastic region, (5) direction of the external load relative to the symmetry elements of the foams; e.g., parallel or perpendicular to the rise direction, (6) shape and aspect ratio of the specimens (height to width), (7) length and location of the measurement base, (8) strain rate, etc. At each combination of the mentioned factors, it would be necessary to produce PU foams in a sufficiently large density range to determine A and b accurately. With A and b known, the value of a modulus or strength can be determined at any density, for which the Equation (6) is valid.

Analysis of the experimental data of Poisson’s ratios and moduli showed that the filled NEOpolyol-380 PU foams produced were nearly isotropic. Then at each concentration η of the filler

E(η) = B(η)*ρ*^c(η)^, (7)

where B(η) and c(η) are parameters. At each concentration of filler, the number of compression specimens in a group was ≤5 and the number of tension specimens was ≤6. The experimentally measured values of E(η) were in a too narrow range of density to determine B(η) and c(η) accurately. It would be necessary to produce the foams in a sufficiently large density range at each concentration of filler and each loading mode. However, due to the technological requirement m_0_ = 250 g ≈ const., the average density of each group of the selected specimens differed little from the common density *ρ*_com_ = 224 kg/m^3^ which was calculated as the average density of the 104 selected compression and tension specimens. The relative difference between *ρ*_com_ and the average density of the selected compression specimens of each group was <3% and of the selected tension specimens, <5%. That permits us to assume c(η) ≈ b and calculate B(η) for each specimen of density *ρ*:

B(η) = E(η)/*ρ*^b^, (8)

where E(η) is the experimentally measured compression modulus. If there are M ≤ 5 selected compression specimens in a group, the average B(η), characterizing the whole group, can be calculated:

(9) $$B_{\mathrm{av}}(\eta )=1/M\sum_{m=1}^{M}{E(\eta )}_{m}/(\rho_{m}{)}^{b}$$

Then, the normalized modulus is calculated as:

E_norm_(η) = B_av_(η)(*ρ*_com_)^b^ (10)

In [9], isotropic neat NEOpolyol-380 PU foams of the same formulation and in the same mold as in the present study were produced. To compare the neat NEOpolyol-380 PU foams produced in the present study and in [9], the values of A and b of neat NEOpolyol-380 PU foams were calculated from the experimental data curves “E-*ρ*” and “σ-*ρ*” in [9], Table 2.

Drawing the b values from Table 2, the average A values were calculated for neat NEOpolyol-380 PU foams for the three groups of selected specimens in the present study, Table 3.

The slight differences in A values can be explained by different batches of chemicals and different shapes of compression specimens. No selection of specimens with similar characteristics of anisotropy was made in [9]. In general, good repeatability of the neat NEOpolyol-380 PU foams was identified, allowing us to assume c(η) ≈ b, to draw the b values from the Table 2 and to calculate the averaged and normalized moduli.

The other mechanical properties were normalized in a similar way. No values of parameters A and b were available for the elongation at break ε_11max_ for neat NEOpolyol-380 PU foams. Therefore, the experimental data of ε_11max_ of typical petrochemical PU foams [1] was combined with the ε_11max_ values acquired in the given investigation for neat NEOpolyol-380 PU foams (the selected tension specimens) and the parameters A and b of the best fitting model function, Equation (6), were determined. The density-independent Poisson’s ratios were not normalized. Averaging of the ν_12_^C^, ν_32_^C^ and ν_12_^T^ values of N1 (N2 and N3) selected specimens from a certain block and at a certain loading mode was carried out to calculate the average Poisson’s ratios at a certain concentration of the filler:

(11) $$\nu {12_{\mathrm{av}}}^{C}=1/N1\sum_{n=1}^{N1}\nu_{12n}^{C},\;\nu {32_{\mathrm{av}}}^{C}=1/N2\sum_{n=1}^{N2}\nu_{32}^{C}\;\mathrm{and}\;\nu {12_{\mathrm{av}}}^{T}=1/N3\sum_{n=1}^{N3}\nu_{12}^{T}.$$

Considering *ρ*_com_ and b as constants, the uncertainty of a normalized modulus was estimated as ΔE = (*ρ*_com_)^b^ΔB_avE_, of a normalized strength as Δσ_max_ = (*ρ*_com_)^b^ΔB_avσ_, of a normalized stress at 10% strain as Δσ_10%_ = (*ρ*_com_)^b^ΔB_avσ_, of a normalized elongation at break as Δε_11max_ = (*ρ*_com_)^b^ΔB_avε_ and of a Poisson’s ratio as Δν, where ΔB_avE_, ΔB_avσ_ and ΔB_avε_ are the standard deviations of B_avE_, B_avσ_ and B_avε_ values and Δν is the standard deviation of Poisson’s ratio values.

### 3.3. Dependence of Mechanical Properties on Concentration of Filler

The dependence of the NEOpolyol-380 PU foams’ averaged and normalized mechanical properties on the concentration of the filler of the selected specimens was analyzed and compared with the same properties calculated for all specimens (the selected + the excluded).

Several other elastic constants of the slightly monotropic NEOpolyol-380 PU foams were calculated at each concentration of filler, based on the linear elasticity theory [29] and using the experimentally determined ones:

E_2_ = E_1_; (12)

(13) $$\frac{E_{3}}{E_{1}}=\frac{\nu_{31}}{\nu_{13}},\;\mathrm{then}\;\nu_{13}=\nu_{31}E_{1}/E_{3}\mathrm{and}$$

ν_21_ = ν_12_, ν_31_ = ν_32_ and ν_23_ = ν_13_. (14)

For strength and elongation at break in compression and in tension, it is valid that:

σ_22max_ = σ_11max_ and ε_22max_ = ε_11max_. (15)

When the selected compression specimens are nearly isotropic, the shear and bulk moduli can be estimated as follows:

(16) $$E_{av}=\frac{2E_{1}+E_{3}}{3},\;\nu_{av}=\frac{1}{3}\left(\nu_{12}+\nu_{32}+\nu_{13}\right),\mathrm{then}\;G=\frac{E_{av}}{2(1+\nu_{av})},\;K=\frac{E_{av}}{3(1-2\nu_{av})}$$

where E_1_, E_3_ and ν_12_, ν_32_ and ν_13_—elastic constants in compression.

## 4. Results and Discussion

### 4.1. XRD Analysis Results

**Figure 3:**
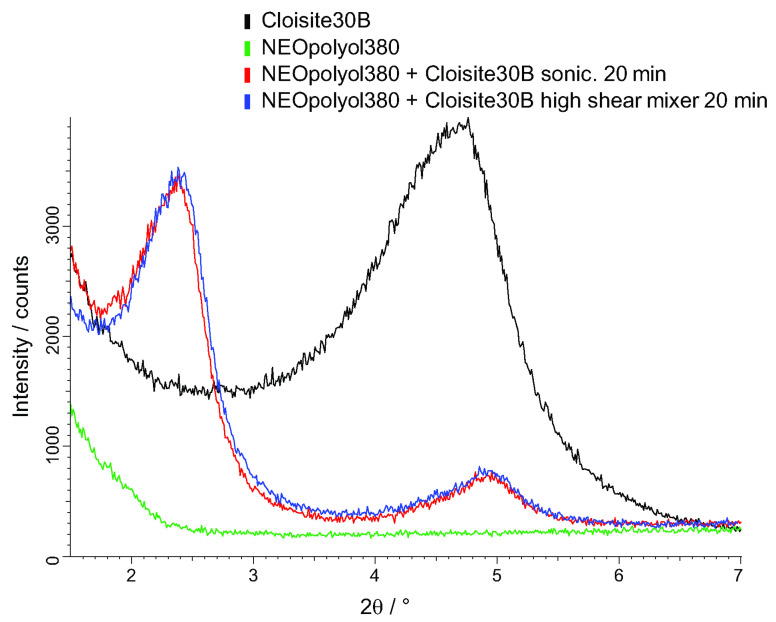
XRD patterns of Cloisite-30B (black), NEOpolyol-380 (green) and a 5% dispersion of Cloisite-30B in NEOpolyol-380 made by sonication for 20 min (red) and high shear mixing for 20 min (blue).

The 5 wt.% Cloisite-30B dispersions made by (a) high shear mixing for 20 min and (b) sonication for 20 min gave similar XRD patterns, as shown in Figure 3, confirming similar efficiency for both methods. The “Cloisite-30B-NEOpolyol-380” dispersions for the production of the filled NEOpolyol-380 PU foams were prepared with high-shear mixing as it is a technically more convenient method (no ultrasound).

Characteristic changes were observed in the diffraction patterns: (1) the angular position of the reflex 001 moved to smaller angles due to penetration of the macro chains into galleries and (2) the intensity of the diffraction peak decreased because of delamination of the nanoclay particles under the action of shear forces. The diffraction peaks of the dispersions shifted to the left, compared to those of pure Cloisite-30B, Figure 3. A change in d-spacing between the planes of the diffraction lattice was identified according to Bragg’s law:

d = nλ/(2sinθ), where the diffraction order n = 1, 2 and 3.(17)

The first diffraction peak shifted from an angle 2θ = 4.75° to 2θ = 2.38°, which corresponded to an increase in d-spacing (001) from 18.6 Å to 37.1 Å. The second diffraction peak corresponded to an angle 2θ ≈ 4.9°. The polyurethane chains grow during polymerization, which facilitates an expansion of the interlayer spacing and an exfoliation of nanoclay platelets in the polymer matrix. The nanoclay Cloisite-30B had not fully exfoliated and intercalation dominated, as indicated by the still visible diffraction peaks.

### 4.2. PU Foam Blocks

The relative difference between the target mass m_0_ = 250 g = const. and actual mass m of each of the 7 NEOpolyol-380 PU foam blocks (244 g ≤ m ≤ 261 g) was estimated as ≤4%; therefore, the technological requirement m = m_0_ = const. was considered as executed.

The density of the neat, free-rise NEOpolyol-380 PU foam of the given formulation, block No. 1, was measured as *ρ*_0_ ≈ 145 kg/m^3^. If the apparent overall density of a block produced in a sealed mold is *ρ*_sm_ ≈ 240 kg/m^3^, then the value of overpressure in the sealed mold p_ov_ = *ρ*_sm_/*ρ*_0_ = 240/145 ≈ 1.7 atm. The PU foams in the sealed mold are produced at a high overpressure, ensuring a nearly isotropic structure.

The average content of closed cells of the neat NEOpolyol-380 PU foam in block No. 1 was measured as 99%.

### 4.3. Density of Specimens

#### 4.3.1. Gradient of Density

The density of section C-a differed little from the density of section C-b for all NEOpolyol-380 PU foam blocks. The relative difference in densities R < 1%; therefore, the gradient of density *ρ*’ = δ*ρ*/δx_2_ along axis OX_2_ was considered as small in sections C-a and C-b. The light microscopy showed that a more rapid increase in density started at a distance ~10–15 mm from the sides of blocks. Due to the square horizontal cross section of the mold, the foam blocks had a fourth order rotational symmetry C_4_ around axis OX_3_, which permitted a zone of highly uniform density (Figure 2, the green rectangles) to be outlined. The C_4_ symmetry led to similar foaming conditions in locations (1) “1 Side” and “5 Side”, (2) “1′ Side’”, “2 Intermediate”, “4 Intermediate” and “5′ Side” as well as (3) “2′ Intermediate” and “4′ Intermediate”.

#### 4.3.2. Density of Compression and Tension Specimens

**Figure 4:**
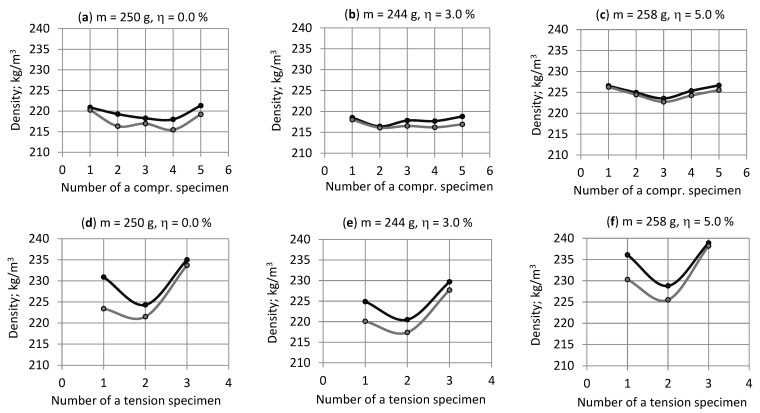
Density of NEOpolyol-380 PU foam specimens from blocks Nos. 1, 6 and 7 (as examples, the density distribution in the blocks Nos. 2, 3, 4 and 5 was similar); concentration of filler η = 0.0, 3.0 and 5.0%: (**a**–**c**) the compression specimens from sections C-a (black) and C-b (gray) and (**d**–**f**) the straight part of tension specimens from sections T-a (black) and T-b (gray).

The average density, standard deviation and coefficient of variation was estimated for compression specimens from section C-a as *ρ*_av_ = 222.3 kg/m^3^ ± 4.1 kg/m^3^ (1.9%) and from section C-b as *ρ*_av_ = 220.8 kg/m^3^ ± 4.4 kg/m^3^ (2.0%). Density was the smallest for specimens from locations 3 and 3′ (“Central”) of sections C-a and C-b. It increased for 1 kg/m^3^–4 kg/m^3^ to the sides of a block (specimens at locations 1, 1′ and 5, 5′), Figure 4a–c.

The average density was estimated for the precursors of the tension specimens from sections T-a *ρ*_av_ = 236.1 kg/m^3^ ± 7.3 kg/m^3^ (3.1%) and T-b 230.7 kg/m^3^ ± 6.6 kg/m^3^ (2.8%). Then the average density was estimated for the straight part of the tension specimens from sections T-a *ρ*_av_ = 230.4 kg/m^3^ ± 5.7 kg/m^3^ (2.5%) and T-b 226.5 kg/m^3^ ± 6.5 kg/m^3^ (2.9%). Density was the lowest for the specimens from locations 2 and 2′ (middle) of sections T-a and T-b. It increased to 4 kg/m^3^–12 kg/m^3^ in the top and bottom of a block in section T-a and to 2 kg/m^3^–13 kg/m^3^ in section T-b, Figure 4d–f.

The density of the straight part of the tension specimens from sections T-a and T-b at locations 2 and 2′ (“Middle”) was in good correlation with the density of the compression specimens from locations 3 and 3′ (“Central”). The relative density differences were ≤3.3%. The average density of the compression specimens from section C-a (C-b) was ~8 kg/m^3^ (~6 kg/m^3^) less than the average density of the tension specimens from section T-a (T-b).

### 4.4. Mechanical Properties of NEOpolyol-380 PU Foams

At all concentrations of the filler, the general character of the “stress-strain” curves in compression and in tension remained similar to that of the neat NEOpolyol-380 PU foams. No stress maximum was detected in compression up to 10%, when loading was terminated. No visible signs of collapse were observed on the side surfaces of the tested specimens. The remaining strain of the compression specimens ≈ 1.5 years after the testing was measured as ≈3% (≈70% of the strain had recovered).

The tension specimens from section T-b had a lower density in the work zone compared to the ends, but for section T-a specimens, the density of the straight part was similar to that at the ends. Consequently, ≈90% of the section T-b specimens broke in the straight zone and ≈40% of the section T-a specimens. All tension specimens exhibited sufficient compression and shear resistance for gripping in the fixtures of the testing machine without supplementary appliances.

### 4.5. Poisson’s Ratios and Density

When gripped in the fixtures of the testing machine, the tension specimens of the light-weight industrial PU foams broke prematurely at the ends. Iron fixtures [13] were glued to the ends of the specimens to facilitate load transfer to the work zone. Stress maximums were detected in compression below 10% and strength in compression was determined as the maximum compressive force divided by the initial cross-section of the specimen; ISO 844:2021(E).

The results of the mechanical testing of the industrial, monotropic PU foams showed (1) similar values of Poisson’s ratios may correspond to different densities of foams and (2) the relationship E_3_/E_1_ ≈ ν_31_/ν_13_ was fulfilled, Table 4. While the moduli E_1_ and E_3_ are dependent on the foams’ density, the ratio E_3_/E_1_ depends only on the anisotropy of the foams’ material.

**Figure 5:**
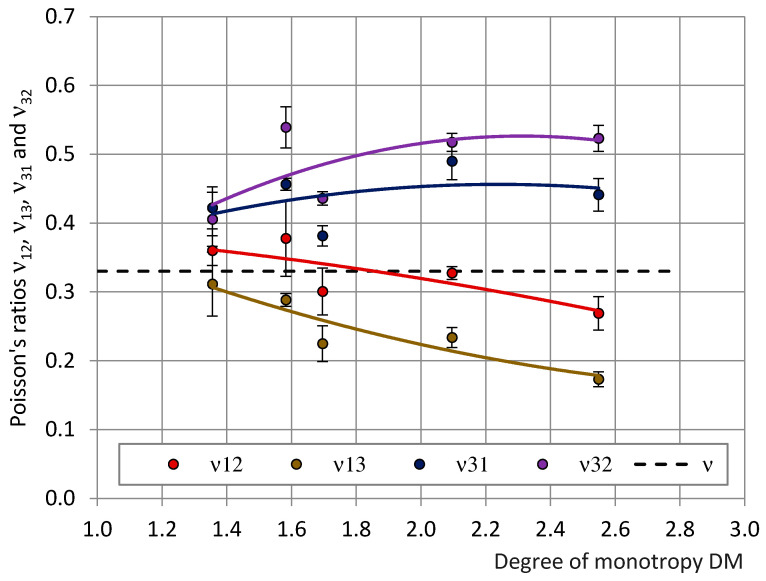
The dependence of Poisson’s ratios ν_12_, ν_13_, ν_31_ and ν_32_ on the degree of monotropy DM of the industrial PU foams. For isotropic PU foams ν = 0.33 (Black dashed line).

The dependence of ν_31_, ν_32_, ν_12_ and ν_13_ on the degree of monotropy DM = E_3_/E_1_ = ν_31_/ν_13_ in compression is given in Figure 5. When DM → 1.0 (isotropic foams), ν_31_, ν_32_, ν_12_ and ν_13_ → ν = 0.33. The coefficients ν_12_ and ν_13_ decreased and coefficients ν_31_ and ν_32_ increased with an increase in DM which can be explained by an increasing orientation of polymeric struts in the rise direction OX_3_ [1,23,30,31]. The experimental data confirmed that Poisson’s ratios depend on the monotropy degree of the foams and do not depend directly on density. Within the limits of the linear elasticity theory, the statement remains valid for any cellular plastics, independent of their particular formulation and/or density [4,22,23].

### 4.6. Selection of the Specimens of NEOpolyol-380 PU Foams

The analysis of the three experimental data sets of Poisson’s ratios: ν_12_ and ν_32_ in compression and ν_12_ in tension showed that the data of eight specimens were not fulfilling the criterion (4) (≈7% of 112 specimens in total). Namely, (a) in the compression data of three specimens: one from location 3 (“Central”), one from location 3′ (“Central”) and one from location 5 (“Side”) and (b) in the tension data of five specimens: one from location 2 (“Middle”) and four from location 3′ (“Top”). These eight specimens are denoted as inappropriate. The data of 104 specimens (≈93% of 112 specimens) fulfilled criterion (4) and these specimens were denoted as appropriate.

Criterion (4) was not fulfilled by the Poisson’s ratios of the compression specimens of the neat foams, block No. 1, the “Central” locations 3 and 3′, Table 5. The mechanical properties of the foams exhibited a slight degree of monotropy, DM:

E_3_ = 138 MPa > E_1_ = 99 MPa; ν_32_ = 0.39 > ν_12_^C^ = 0.23; σ_33(10%)_ = 3.6 MPa > σ_11max_ = 3.1 MPa and DM = E_3_/E_1_ = 138 MPa/99 MPa ≈ 1.39. (18)

Criterion (4) was not fulfilled by the Poisson’s ratios of the “Side” specimen from location 5 of block No. 4, Table 6. Its mechanical properties exhibited a slight monotropy:

E_3_ = 160 MPa > E_1_ = 127 MPa; ν_32_ = 0.39 > ν_12_ = 0.30; DM = E_3_/E_1_ = 160 MPa/127 MPa ≈ 1.26. (19)

If the mold happens to be placed askew during foaming, one or two of its corners are located above the others. However, the liquid reacting mixture maintains a horizontal level due to the action of gravitation. The free space, available for foaming above the level of the liquid, is determined by the distance to the lid, which is greatest in the elevated corner. Foam in the corner location 5 (“Side”) has space to rise to a higher degree of monotropy than in the other locations.

The selection of tension specimens from sections T-a and T-b identified—in four cases out of five—that criterion (4) was not fulfilled by the Poisson’s ratios of the specimens from the “Top” location 3′ of section T-b, blocks Nos. 1, 2, 3 and 7, Table 7. In this location, the straight part of the tension specimen was at the top of the rising foam, the closest to the CGRH, and the comparatively small values of ν_12_ suggested a medium degree of monotropy.

The inappropriate value ν_12_^T^ = 0.34 for the specimen from the “Middle” location of section T-a, block No. 2, Table 7 may have been caused by a local foaming defect.

Of the eight inappropriate specimens, six (75%) belonged to the No. 1 block (the neat, unfilled block; η = 0.0%) and the No. 2 block (the lowest non-zero concentration η = 0.25%) which had the lowest viscosity of the liquid reacting mixture and the highest speed of rise of the mixture before reaching the lid of the mold (Figure 1). As a result, the monotropy degree of the foams in blocks Nos. 1 and 2 was the highest. The neat foams were the reference material when evaluating the effect of filling (Equations (23) and (24)); therefore, it was important to estimate their mechanical properties precisely. On the other hand, of the eight inappropriate specimens, six were from the “Central” and “Top” locations. At the free-rise stage, the central part of a block rises with the highest speed, because at the sides of a block, the reacting mixture sticks to the walls.

The average Poisson’s ratios of the selected specimens over all the concentrations of filler were calculated in compression and in tension:

ν_32av_^C^ = 0.34 ± 0.02 (4%), ν_12av_^C^ = 0.32 ± 0.02 (5%) and ν_12av_^T^ = 0.31 ± 0.01 (5%). (20)

In general, when the values of Poisson’s ratio are in a range 0.30 ≤ ν ≤ 0.33, PU foams exhibit an isotropy of structure and mechanical properties [1,2,4]. The values in Equation (20) identify a slight monotropy:

ν_32av_^C^ > ν_12av_^C^, ν_32av_^C^ > ν_12av_^T^ and ν_12av_^C^ ≈ ν_12av_^T^. (21)

With the averaged and normalized moduli E_3av_^C^ and E_1av_^C^ determined, the average monotropy degree of the 67 selected compression specimens was estimated:

(22) $${{\mathrm{DM}}_{\mathrm{av}}}^{C}=1/7\sum_{m=1}^{7}E_{3avm}^{C}/E_{1avm}^{C}\;=\;1.03\pm 0.04\;(4\%).$$

The value of DM_av_^C^ identified nearly isotropic foams and was in good correspondence with the microscopy data of the same NEOpolyol-380 PU foam blocks [32]. Since the selected compression specimens are nearly isotropic, the shear and bulk moduli were estimated according to the Equations (16).

When the values of the parameters in criterion (4) were set to δ_1_ = δ_2_ = δ_3_ = 0.05 (5%), the number of inappropriate specimens increased, but the calculated mechanical properties varied insignificantly; therefore, the values δ_1_ = δ_2_ = δ_3_ = 0.1 (10%) were considered as sufficient for the given data sets.

### 4.7. Dependence of the Mechanical Properties on Concentration of Filler

**Figure 6:**
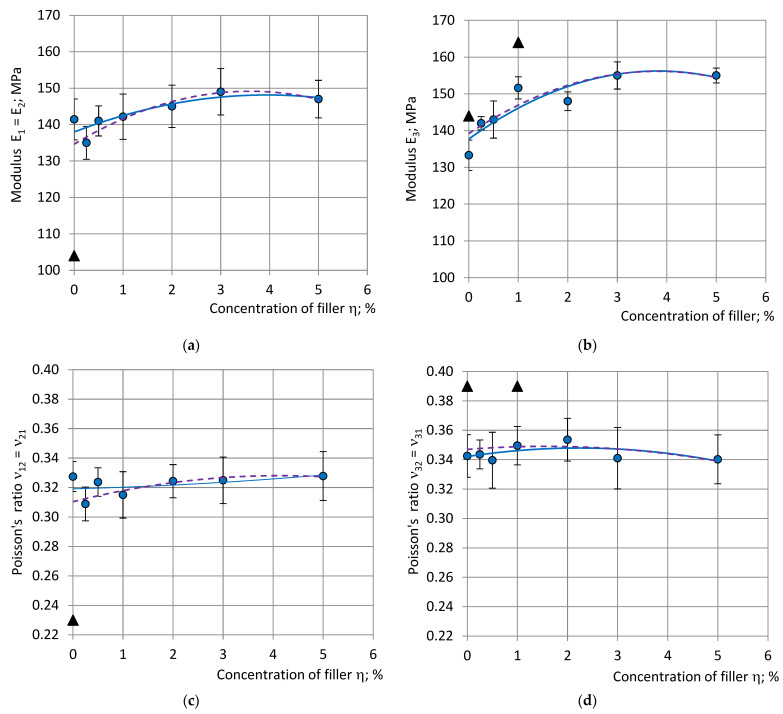

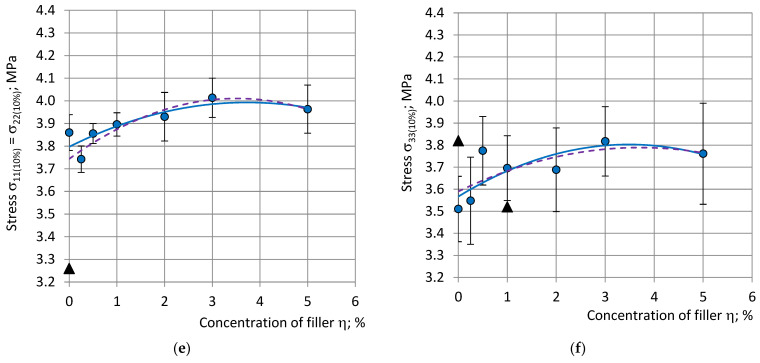
Moduli (**a**) E_1_ = E_2_ and (**b**) E_3_, Poisson’s ratios (**c**) ν_12_ = ν_21_ and (**d**) ν_31_ = ν_32_, stress at 10% strain (**e**) σ_11(10%)_ = σ_22(10%)_ and (**f**) σ_33(10%)_ in compression, with dependence on concentration of filler: blue—data and trendlines of the selected specimens; the black markers—data points of the excluded specimens; and violet—trendlines for data of all specimens.

**Figure 7:**
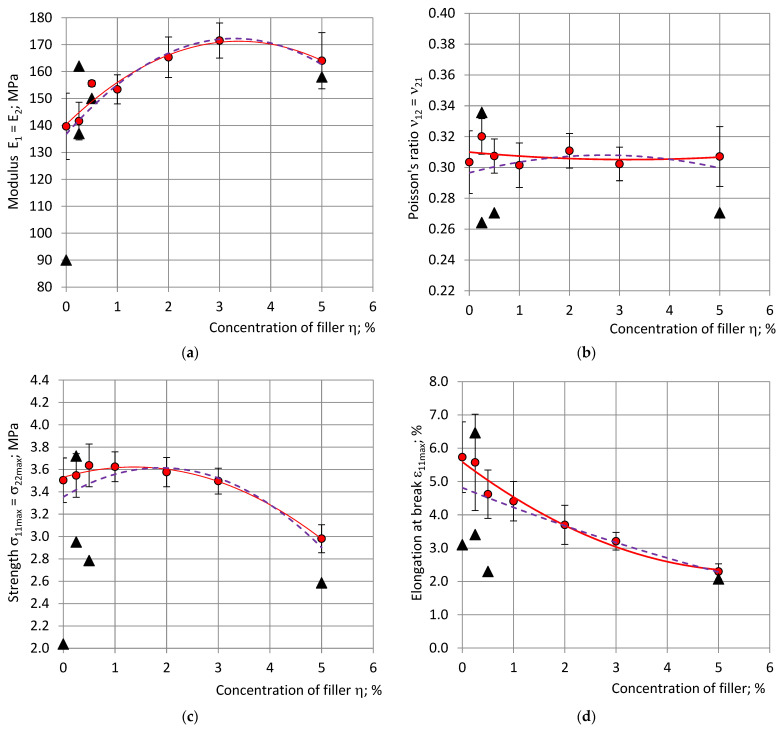
(**a**) Moduli E_1_ = E_2_, (**b**) Poisson’s ratio ν_12_ = ν_21_, (**c**) strengths σ_11max_ and (**d**) elongation at break ε_11max_ in tension, with dependence on concentration of filler: red—data and trendlines of the selected specimens; the black markers—data points of the excluded specimens; and violet—trendlines for data of all specimens.

**Figure 8:**
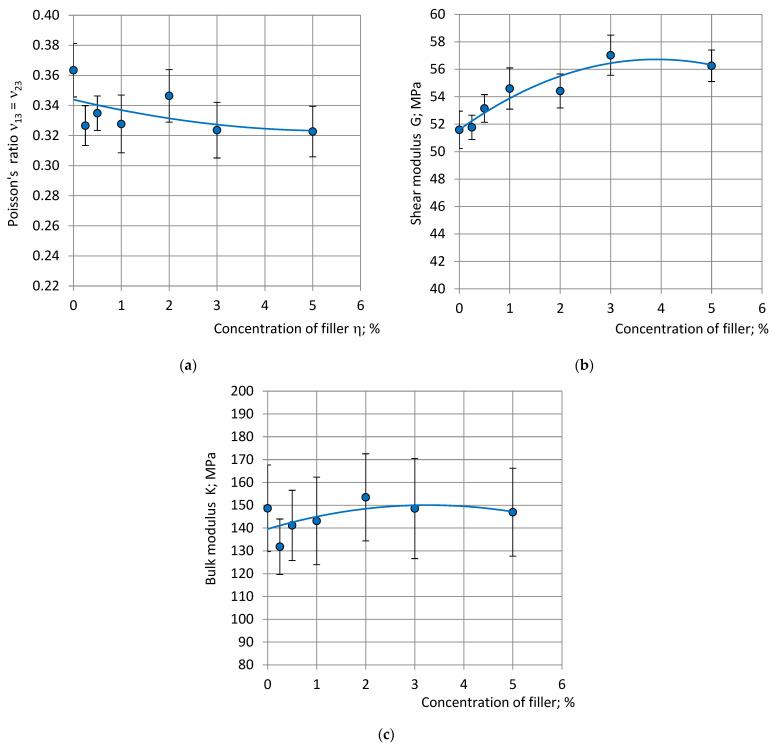
The other calculated elastic constants (**a**) Poisson’s ratios ν_13_ = ν_23_ in compression, (**b**) shear modulus and (**c**) bulk modulus with dependence on concentration of filler (the selected specimens).

The elastic moduli, strength and stress at 10% strain, averaged and normalized at density *ρ*_com_ = 224 kg/m^3^, and the averaged Poisson’s ratios of the selected specimens are given in Figure 6 and Figure 7 (continuous curves) for compression and tension, together with the trendlines of the results calculated for all specimens (dashed curves). The other calculated elastic constants (shear and bulk moduli) are given in Figure 8.

It can be seen that some data of the excluded specimens differs from the averaged data of the selected specimens ≈1.5–2 times; e.g., ν_12_ in compression and E_1_ in tension. The relative change R in moduli, stress at 10% strain and elongation at break due to filling was estimated according to the trendlines, e.g., for modulus E_1_ in compression:

R(E_1_) = ΔE_1_/E_1_ = (E_1fil_ − E_10_)/E_10_; (23)

where E_1fil_—the highest E_1_ value of the filled foams and E_10_–E_1_, the value of neat foams. With an increase in the concentration of filler η compression moduli E_1_, E_3_ and stress at 10% strain σ_11(10%)_, σ_33(10%)_ increased and reached their maximum at η = 3 … 4%. Modulus E_1_ increased by ≈7%, E_3_ by ≈13%, σ_11(10%)_ by ≈5% and σ_33(10%)_ by ≈6%. The calculated shear modulus G increased by ≈9% at η = 3% and bulk modulus by ≈7%. A further increase in concentration to 5% led to a slight decrease in the mechanical properties.

In the tension modulus, E_1_ increased by ≈22% at η = 3 … 4% and decreased by 5%; the strength in tension σ_11max_ decreased by ≈16% and the elongation at break by ≈58% at η = 5.00%, compared to the neat foams. Poisson’s ratios remained nearly constant at all concentrations, both in compression and in tension: ν_32av_^C^ ≈ 0.34, ν_12av_^C^ ≈ 0.32 and ν_12av_^T^ ≈ 0.31, which confirmed the isotropic structure of the foams.

The selection of specimens mainly influenced the values of the mechanical properties of the neat foams and the foams with the low concentrations of filler: η = 0.25, 0.50 and 1.00%, see Figure 5 and Figure 6. The neat PU foam is a reference material for the estimation of the effect of filling; therefore, an accurate evaluation of its mechanical properties is crucial. The relative adjustment, RA, caused by the relative change R in the selection of specimens, was calculated as:

RA = (R_all_ − R_sel_)/R_all_; (24)

where R_all_ is the relative change when data of all specimens is taken into account and R_sel_—the same for data of the selected specimens, Table 8.

The stiffness of the filled PU foams was higher than that of neat foams both in compression and tension. The stiffness of nanoclay platelets in compression is estimated as 175–265 GPa [33] and that of dry clay particles as 6.2 GPa [34] which is higher than the stiffness of the PU matrix at 2500 MPa [1,21,28]. Then, the rule of mixture predicts an increased stiffness for a “PU foam-nanoclay” composite. The increase in the stress at 10% strain in compression might be attributed to the creation of multiple crack sites and branches by the nanoparticles, which delay the propagation of fracture [12,35,36,37].

In tension, the decrease in strength and elongation at break might be caused by a weakness of the “PU–nanoclay” interface in tension; thus, the nanoclay platelets act as the initiators of a crack. The platelets, when dispersed uniformly into the polymer, produce a huge number of interface regions as compared to microcomposites and the interphase can become a dominating factor determining the properties of the nanocomposite.

## 5. Conclusions

As estimated, ≈93% of the PU foam specimens fulfilled the criterion of similar anisotropy characteristics and ≈7% failed to fulfill it. Specimens from “Central” and “Top” locations did not fulfill the mentioned criterion because of the highest speed of PU foams’ rise in the centre of the blocks, close to the CGRH and relatively far from the sides of the mold. The speed of rise being the highest in the centre of a block cannot be avoided without changing the technology. It is a systematic, predictable effect. Several specimens from the “Side” locations did not fulfill the criterion because of a technological flaw—a mold placed askew at foaming. It was a random, unpredictable effect which could be avoided by placing the mold in a precisely horizontal position.

The influence of the nanoclay filler Cloisite-30B on the mechanical properties of NEOpolyol-380 PU foams was estimated at filler concentrations of 0.00, 0.25, 0.50, 1.00, 2.00, 3.00 and 5.00% by the relative change in the foams’ mechanical properties due to filling relative to the mechanical properties of unfilled, neat foams. It was shown that the selection of the mechanical testing specimens of foams, according to the criterion of similar anisotropy characteristics, reduced the influence of the structural anisotropy variations on the estimation of the foams’ mechanical properties. The relative adjustment, provided in the estimation of the NEOpolyol-380 PU foams’ mechanical properties by the selection of specimens, was ≈10–30%.

The benefit for practical applications of the criterion of similar anisotropy characteristics lies in a comparatively simple, efficient method for identifying and excluding mechanical testing specimens of PU foams with an unacceptably different structural anisotropy from those with a similar anisotropy. Further processing of the testing data of the excluded specimens can be avoided to save time and resources. In principle, the elaborated methodology is applicable to PU foams produced both in closed and open molds.

The filling of rigid NEOpolyol-380 PU foams with a density of 215–240 kg/m^3^ with the nanoclay filler Cloisite-30B up to 5 wt.% from the mass of filled reacting mixture moderately improved the mechanical properties of the foams in compression and tension, due to the nanoclay not being fully exfoliated, as suggested by the XRD patterns. It may have been caused by the reduced efficiency of the organic modification of the Cloisite-30B filler’s surface, because the production year of the filler was the year 2010, but it was added to the NEOpolyol-380 PU foams in the year 2020.

More research is necessary on normalization of the mechanical properties of anisotropic PU foams to a common density.

## Figures and Tables

**Table 1:** NEOpolyol-380 PU foams’ formulation.

Formulation of Polyol (pbw) *		
1	Recycled APP NEOpolyol-380	80.0
2	Cross-linking agent, Lupranol 3422	20.0
3	Flame retardant, TCPP	20.0
4	Blowing agent, water	1.0
5	Reactive catalyst, PC CAT NP-10	1.6
6	Surfactant, NIAX Silicone L6915	2.0
Polyisocyanate (pbw)		193
Characteristics of Formulation		
1	Recycled materials in PU foams (%)	15
2	Isocyanate index	160
Technological Parameters		
1	Cream time (s)	25
2	String time (s)	45
3	Tack-free time (s)	60
4	Foaming end time (s)	60

* Parts by weight per hundred parts of polyol.

**Table 2:** Parameters A and b of neat NEOpolyol-380 PU foams; densities 40 kg/m^3^ ≤ *ρ* ≤ 600 kg/m^3^.

Mechanical Property	Compression		Tension	
	A (MPa·kg·m^−3^)^−b^)	b	A (MPa·(kg·m^−3^)^−b^)	b
Modulus E	0.0109	1.68	0.0110	1.75
Stress σ _ 10% _ /strength	0.00030	1.75	0.0035	1.30

**Table 3:** Parameters A and b of neat NEOpolyol-380 PU foams.

Mechanical Property	Average Density; kg/m^3^	Compression		Average Density; kg/m^3^	Tension	
		A_av_ (MPa·(kg·m^−3^)^−b^)	b		A_av_ (MPa·(kg⋅m^−3^)^−b^)	b
Modulus E_1_	218	0.0159	1.68	227	0.0108	1.75
Modulus E_3_	220	0.0150	1.68	-	-	-
Stress σ_11(10%)_/strength	218	0.00030	1.75	227	0.0031	1.30
Stress σ_33(10%)_/strength	220	0.00027	1.75	-	-	-

**Table 4:** The physical and mechanical properties of the industrial PU foams (*ρ*_pol_ = 1196 kg/m^3^ and γ = *ρ*/*ρ*_pol_).

Property/Block No.	1	2	3	4	5
(1) Compression parallel to the axis OX_1_					
Density *ρ* (kg/m^3^)	33	39	56	76	82
Relative density γ (%)	2.8	3.2	4.6	6.3	6.9
Modulus E_1_ (MPa)	4.3	5.6	9.7	22.5	7.9
Poisson’s ratio ν_12_	0.27	0.38	0.33	0.36	0.30
Poisson’s ratio ν_13_	0.17	0.29	0.23	0.31	0.22
Strength σ_11max_ (MPa)	0.17	0.19	0.32	0.60	0.66
Strain ε_11max_ (%)	5.4	6.5	5.3	5.4	4.4
(2) Compression parallel to the axis OX_3_					
Density *ρ* (kg/m^3^)	32	38	55	75	81
Relative density γ (%)	2.7	3.2	4.6	6.3	6.8
Modulus E_3_ (MPa)	9.3	9.4	19.2	31.5	28.7
Poisson’s ratio ν_31_	0.44	0.46	0.49	0.42	0.38
Poisson’s ratio ν_32_	0.52	0.54	0.52	0.41	0.44
Strength σ_33max_ (MPa)	0.28	0.26	0.52	0.72	0.73
Strain ε_33max_ (%)	4.1	4.4	3.3	3.1	3.0
Characteristics of anisotropy					
Ratio E_3_/E_1_	2.2	2.0	1.7	1.4	1.6
Ratio ν_31_/ν_13_	2.5	2.1	1.6	1.4	1.7
(3) Tension parallel to the axis OX_3_					
Density *ρ* (kg/m^3^)	34	34	51	76	77
Relative density γ (%)	2.8	2.9	4.2	6.4	6.5
Modulus E_3_ (MPa)	11.1	15.3	26.3	37.9	40.2
Poisson’s ratio ν_31_	0.65	0.64	0.56	0.46	0.46
Poisson’s ratio ν_32_	0.58	0.66	0.65	0.50	0.50
Strength σ_33max_ (MPa)	0.46	0.46	0.82	0.96	0.99
Elongation at break ε_33max_ (%)	8.7	7.8	6.9	3.7	1.0

**Table 5:** Mechanical properties of the inappropriate compression specimens; in gray (the neat PU foam block No. 1).

Section C-a					Section C-b				
Number of Specimen and Location	Density *ρ* (kg/m^3^)	Stiffness E_3_ (MPa)	Poisson’s Ratio ν_32_	Stress σ_33(10%)_ (MPa)	Number of Specimen and Location	Density *ρ* (kg/m^3^)	Stiffness E_1_ (MPa)	Poisson’s Ratio ν_12_	Stress σ_11(10%)_ (MPa)
1 Side	221	130	0.35	3.4	1′ Side	220	143	0.34	3.8
2 Intermediate	219	130	0.36	3.3	2′ Intermediate	216	136	0.33	3.7
3 Central	218	138	0.39	3.6	3′ Central	217	99	0.23	3.1
4 Intermediate	218	132	0.33	3.6	4′ Intermediate	215	130	0.31	3.5
5 Side	221	125	0.33	3.3	5′ Side	219	130	0.33	3.8

**Table 6:** Mechanical properties of the inappropriate compression specimens; in gray (PU foam block No. 4).

Section C-a					Section C-b				
Number of Specimen and Location	Density *ρ* (kg/m^3^)	Stiffness E_3_ (MPa)	Poisson’s Ratio ν_32_	Stress σ_33(10%)_ (MPa)	Number of Specimen and Location	Density *ρ* (kg/m^3^)	Stiffness E_1_ (MPa)	Poisson’s Ratio ν_12_	Stress σ_11(10%)_ (MPa)
1 Side	221	151	0.36	3.5	1′ Side	220	135	0.30	3.8
2 Intermediate	219	143	0.34	3.5	2′ Intermediate	219	144	0.32	3.8
3 Central	221	150	0.36	3.8	3′ Central	217	134	0.32	3.6
4 Intermediate	221	147	0.34	3.6	4′ Intermediate	217	141	0.34	3.7
5 Side	221	160	0.39	3.4	5′ Side	219	127	0.30	3.7

**Table 7:** Mechanical properties of the inappropriate tension specimens; in gray (PU foam block No. 1, 2, 3 and 7).

	Section T-a					Section T-b				
Block Number No.	Number of Specimen and Location	Density *ρ* (kg/m^3^)	Stiffness E_1_ (MPa)	Poisson’s Ratio ν_12_	Strength σ_11max_ (MPa)	Number of Specimen and Location	Density *ρ* (kg/m^3^)	Stiffness E_1_ (MPa)	Poisson’s Ratio ν_12_	Strength σ_11max_ (MPa)
1	1 Bottom	231	159	0.32	3.9	1′ Bottom	223	140	0.29	3.6
	2 Middle	224	146	0.30	3.5	2′ Middle	222	116	0.28	3.2
	3 Top	235	155	0.33	3.6	3′ Top	234	97	0.18	2.2
2	1 Bottom	232	144	0.31	3.5	1′ Bottom	225	142	0.32	3.8
	2 Middle	227	156	0.34	3.8	2′ Middle	223	138	0.33	3.5
	3 Top	236	165	0.32	3.8	3′ Top	234	148	0.26	3.1
3	1 Bottom	228	161	0.31	3.8	1′ Bottom	219	150	0.31	3.6
	2 Middle	221	151	0.32	3.5	2′ Middle	217	146	0.29	3.7
	3 Top	230	163	0.32	3.5	3′ Top	230	157	0.27	2.9
7	1 Bottom	236	180	0.30	3.2	1′Bottom	230	165	0.29	3.1
	2 Middle	229	182	0.33	3.2	2′Middle	226	152	0.29	2.8
	3 Top	239	194	0.33	3.3	3′ Top	232	176	0.27	2.8

**Table 8:** Mechanical properties of PU foams (the selected specimens) and the relative adjustment RA (%).

Mechanical Property	Neat PU Foams	Conc. of Filler η (%)	Filled PU Foams	Relative Change R (%)		Relative Adjustment RA (%)
				All Specimens	Selected Specimens	
(1) Compression						
Modulus E_1_ (MPa)	138	3	148	10	7	−30
Modulus E_3_ (MPa)	139	3	156	12	13	8
Stress σ_11(10%)_ (MPa)	3.8	3	4.0	7	5	−29
Stress σ_33(10%)_ (MPa)	3.6	3	3.8	5	6	20
(2) Tension						
Modulus E_1_ (MPa)	139	3	170	26	22	−15
Strength σ_11max_ (MPa)	3.5	5	3.0	−3	−16	16
Elongation at break ε_11max_ (%)	5.8	5	2.3	−53	−58	10

## Data Availability

Data is contained within the article.
